# Virtual walking therapy in neuropathic spinal cord injury pain: a feasibility study

**DOI:** 10.1038/s41394-024-00667-w

**Published:** 2024-07-31

**Authors:** Gunther Landmann, Marina Aerni, Roger Abächerli, Mario Ernst, André Ljutow, Karina Ottiger-Böttger

**Affiliations:** 1 Neurology, Swiss Paraplegic Centre, Nottwil, Switzerland; 2 Swiss Paraplegic Research, Nottwil, Switzerland; 3 Institute of Physiotherapy, School of Health Professions, Zurich University of Applied Sciences, Winterthur, Switzerland; 4 Lucerne University of Applied Sciences and Arts (HSLU), Horw, Switzerland; 5 Centre for Pain Medicine, Swiss Paraplegic Centre, Nottwil, Switzerland

**Keywords:** Pain management, Quality of life, Outcomes research

## Abstract

**Study design:**

A feasibility study.

**Objectives:**

Chronic neuropathic pain is a prevalent comorbidity in patients with spinal cord injury (SCI), and current medical treatments remain unsatisfactory. New developments as virtual walking are emerging which has been established and further developed at our centre. This study aims to investigate the feasibility of our virtual walking setup in a small group of SCI patients.

**Setting:**

The study was conducted at the Swiss Paraplegic Centre in Nottwil, Switzerland.

**Methods:**

Four patients aged 22 to 60 years were observed during and after therapy. Three had complete paraplegia (levels Th4–Th8) with neuropathic at- and below-level pain, while one had incomplete paraplegia (Th10) with at-level pain. The primary outcome measured was satisfaction with acceptance of and adherence to virtual walking therapy, alongside suggestions for therapy improvements. Additionally, patients kept a pain diary and pain drawings to measure the extent of pain distribution and intensity before and after therapy. Therapy schedules included either two sessions per week for five weeks or five sessions per week for two weeks.

**Results:**

There was a sound satisfaction and good acceptance amongst participants. Support, duration, and number of sessions were perceived well and acceptable. Pain as a secondary outcome did not change during or after therapy in all but one patient which improved in pain intensity, pain quality as well as pain distribution.

**Conclusion:**

Results suggest that our virtual walking setting is a feasible tool that should be further studied in patients with SCI-related chronic neuropathic pain.

## Introduction

Chronic pain is highly prevalent in patients after spinal cord injury (SCI) [1]. The *International Spinal Cord Injury Pain Classification* distinguishes between nociceptive, neuropathic, other, and unknown pain [2]. Point prevalence of chronic neuropathic spinal cord injury pain (SCIP) in SCI is 56% [3] and a recent study of the Swiss SCI population showed that 73% suffer from chronic pain [4]. Despite the high prevalence of SCIP, treatment is still challenging. Recent reviews conclude that there is a lack of evidence for the impact of pharmacological and non-pharmacological treatments [5]. Moreover, medical treatment comes with many side effects [5]. This might be because the underlying biology of SCIP and mechanisms that lead to chronification of pain are not fully understood. One model that may be relevant is the cortical model of pathological pain [6]. This model suggests that a disrupted cortical proprioceptive representation underpins the pain which arises due to a mismatch between motor output and sensory feedback. Therefore, an innovative therapy called virtual walking (VW) has been further developed based on the work of Moseley [7] and the principles of mirror therapy.

The aim of the study is: (1) to assess the feasibility of VW in a small group of patients with SCIP in a clinical setting, (2) to investigate satisfaction with, acceptance of, and adherence to the innovative therapy, and (3) to get patient’s feedback about their experiences and potential improvements to the setup.

## Methods

### Subjects

Patients with SCIP were recruited from July to September 2019. Inclusion criteria: a minimum age of 18 years; SCI since at least a year; at- or below-level SCIP in lower extremities or trunk for at least three months; ability to draw with a pen and German language skills. Exclusion criteria: any neurological, psychological, or cognitive disorder that lead to mandatory hospitalization; epilepsy and pregnancy in women. The study protocol was reviewed by the corresponding ethic commission on 28th June 2019 and it was decided that there is no need for ethical approval. Nevertheless, the ethic commission noted that all general ethical considerations were fulfilled. All patients were screened by a team consisting of a neurologist, a physical therapist and a psychologist. Study inclusion was decided in a group meeting. All patients provided written informed consent before participating in the study.

### Preparatory phase

Before starting the therapy, all participants completed a four-week preparatory phase in which they trained their ability to discriminate between right and left body side. Participants had to download the recognise app of the noigroup. With the app each participant was shown pictures of right and left extremities and had to decide which body side was shown. It was suggested that as an essential component of graded motor imagery treatment [8] this might improve the efficacy of the therapy.

### Virtual walking

**Fig. 1:**
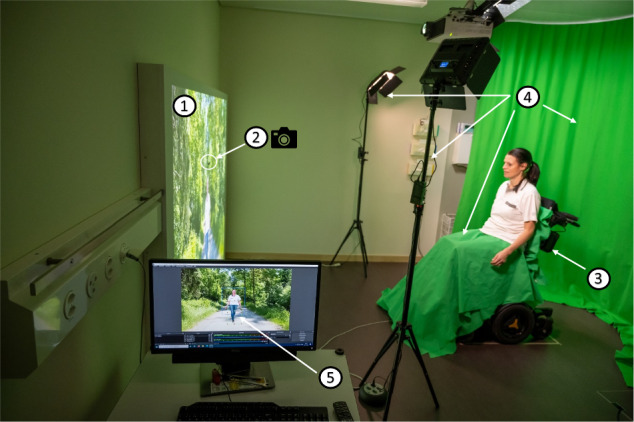
Clinical setting of virtual walking. The setting includes (1) a big projection screen, (2) a built-in camera to film the patient, (3) a modified wheelchair, which is tilting in a horizontal plane and thus mimicking the movement of the pelvis during walking, (4) a green screen setup consisting of a green curtain, a green towel to cover the wheelchair and lower body of the patient and a lighting assembly as well as (5) a computer with sophisticated software to create the customized visual illusion of the walking patient by adding walking legs to the upper body of the patient captured by the camera.

In collaboration with the Lucerne University of Applied Sciences and Arts (HSLU), Horw, Switzerland, a new therapy setting was designed. The setting includes (1) a big projection screen (180 m h, 280 m b), (2) a built-in camera (Realsense^TM^ Depth Camera D435) to film the patient, (3) a modified wheelchair, positioned at a distance of two meters from the screen and tilting in a horizontal plane to the right and left side by 2° mimicking the physiological movement of the pelvis during walking, (4) a green screen setup consisting of a green background, a green towel to cover the wheelchair and patient´s lower body and a lighting assembly and (5) a computer with sophisticated software to create the customized visual illusion of the patient walking by adding walking legs (legs filmed from healthy male and female participants) to the upper body of the patient captured by the camera. This setting enables patients to see themselves walking through a forest from a third-person perspective (Fig. 1). VW therapy was conducted with a schedule of (1) two sessions per week, five weeks in total, or (2) five sessions per week, two weeks in total. Two patients were treated with schedule (1) and two patients with schedule (2).

### Patient reported outcome measures

As primary outcome measure, feasibility was evaluated by asking patients to rate authenticity, satisfaction, willingness to proceed, support, duration, number of sessions and acceptance of the therapy after each week of therapy using a visual analog scale (VAS) ranging from 0 to 100 mm (questionnaire shown in Supplementary Fig. 1). This feasibility questionnaire was developed on the basis of a template from the national association for quality development in hospitals and clinics and extended by additional questions about the acceptance of the duration and frequency of the intervention [9]. Furthermore, all patients underwent a semi-structured feedback interview including the following aspects: (1) satisfaction with and acceptance of therapy, (2) change in pain, (3) positive aspects of therapy, (4) negative aspects of therapy, and (5) therapy improvement suggestions.

In addition, patients were asked to fill in a daily pain diary three weeks before, during, and three weeks after VW therapy.

As secondary outcome measures, participants were asked to fill in self-administered questionnaires to assess pain-related data: Mainz pain staging system (MPSS) which defines the grade of chronification of pain [10]. This 11-item questionnaire assigns pain into one of three possible stadium of chronification. The Graded Chronic Pain Scale (GCPS) was assessed for pain severity which consists of seven questions related to pain intensity with three items, pain-related disability with three items, and the number of disability days with one item [11]. Patient Global Impression of Change (PGIC) was conducted after completion of the virtual walking sessions. PGIC reflects patient’s belief about the efficacy of treatment measured using a seven-point rating scale from much better to much worse [11].

Psychological comorbidities were measured by the Depression, Anxiety, and Stress Scale (DASS). The DASS contains three subscales for depression, anxiety, and stress with seven items each [12]. Catastrophic thinking related to pain experience was rated with the Pain Catastrophizing Scale (PCS) [13].

General well-being was assessed with the Spinal Cord Injury Quality of Life Basic Data Set (SCI-QoL-BDS). The questionnaire contains three variables for general quality of life, physical and psychological health [14].

## Results

### Patients

This feasibility study included four paraplegic patients with SCIP who participated in the VW therapy. Ssocio-demographic and clinical data are shown in Table 1. Ages ranged from 22 to 60 years, one participant was female, time since injury and pain duration ranged from 1.5 to 37 years. Half of the patients suffered from at-level SCIP, the other half from below-level SCIP and all patients had a lesion at the thoracic spine level. The grade of impairment was complete in all but the female patient, who was graded as incomplete and exclusively showed spasticity symptoms.

**Table 1:** Socio-demographic and clinical characteristics of patients.

Patient	Age	Gender	Time since injury (years)	Pain duration (years)	Pain type (SCIP)	Spinal level of lesion	AIS	Spasticity
VW-1	22	female	1.5	1.5	At-level	Th10	C	Yes
VW-2	39	male	4	4	Below-level	Th7	A	No
VW-3	60	male	37	37	At-level	Th11	A	No
VW-4	45	male	6	6	Below-level	Th4	A	No

VW-1-4: Patient code.

*AIS* American Spinal Injury Association Impairment Scale, *SCIP* spinal cord injury pain.

### Patient reported outcome measures

#### Feasibility evaluation questionnaire

**Fig. 2:**
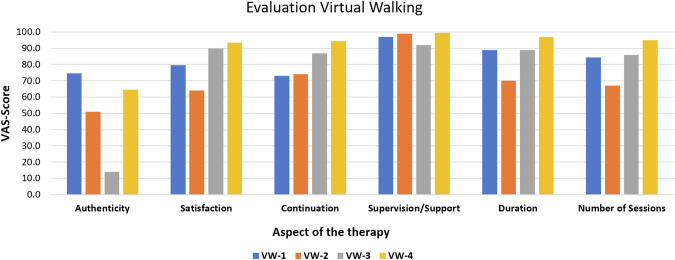
Results from the feasibility evaluation questionnaire. Patients rated six aspects of the therapy with a VAS score (100 mm line). Patients were asked, how authentic their experience with the virtual walking was (Authenticity), how satisfied they were with the therapy (Satisfaction), if they would continue the therapy if they could (Continuation), how they perceived the supervision and the support by the therapist (Supervision/Support), if they were happy with the overall duration of the therapy (Duration) and if the number of single sessions was appropriate (Number of Sessions). Higher scores indicate greater satisfaction or greater agreement with the single statement or question. VW-1-4: Patient code.

The median VAS-scores from the six feasibility items are illustrated and summarized in Fig. 2. “Authenticity” was scored very heterogeneously with values ranging from 14 to 74.5. “Satisfaction” reached moderate values between 64 to 93.5. More consistent results were obtained for “continuation” representing willingness (73 to 94) “duration” of therapy sessions (70 to 97) and “number of sessions” (67 to 95). “Supervision/support” reached the highest values lying between 92 and 100,

#### Semi-structured interview

The results of the semi-structured interview are not shown in detail. A short summary is described as follows:

### Satisfaction/acceptance

In this case, the perspective was criticized due to seeing oneself from the third-person perspective instead of first-person perspective.

### Positive aspects

In some patients, strong memories were evoked during therapy. They imagined how it felt to walk through the forest before the SCI. An improvement in psychological well-being as well as that the intervention was a successful diversification in patient’s therapy schedules were reported. One patient reported that many areas of daily living were positively influenced by the therapy and another patient stated that the therapy was really motivating.

### Negative aspects

Most of the mentioned negative aspects concerned the perspective as well as the environment which was perceived as distracting and thus hindered full immersion into the virtual environment. It was suggested that virtual reality goggles might be more suitable regarding perspective and environment.

One person mentioned a very long sleep period after the third treatment session as a possible side effect and that there was a feeling like a blackout or memory lapse after waking up.

### Suggestions for improvement

Two suggestions for improvement were already adapted during the treatment period. The first was to start the pelvic movement and video at the same time.

#### Pain diary and pain drawing

**Fig. 3:**
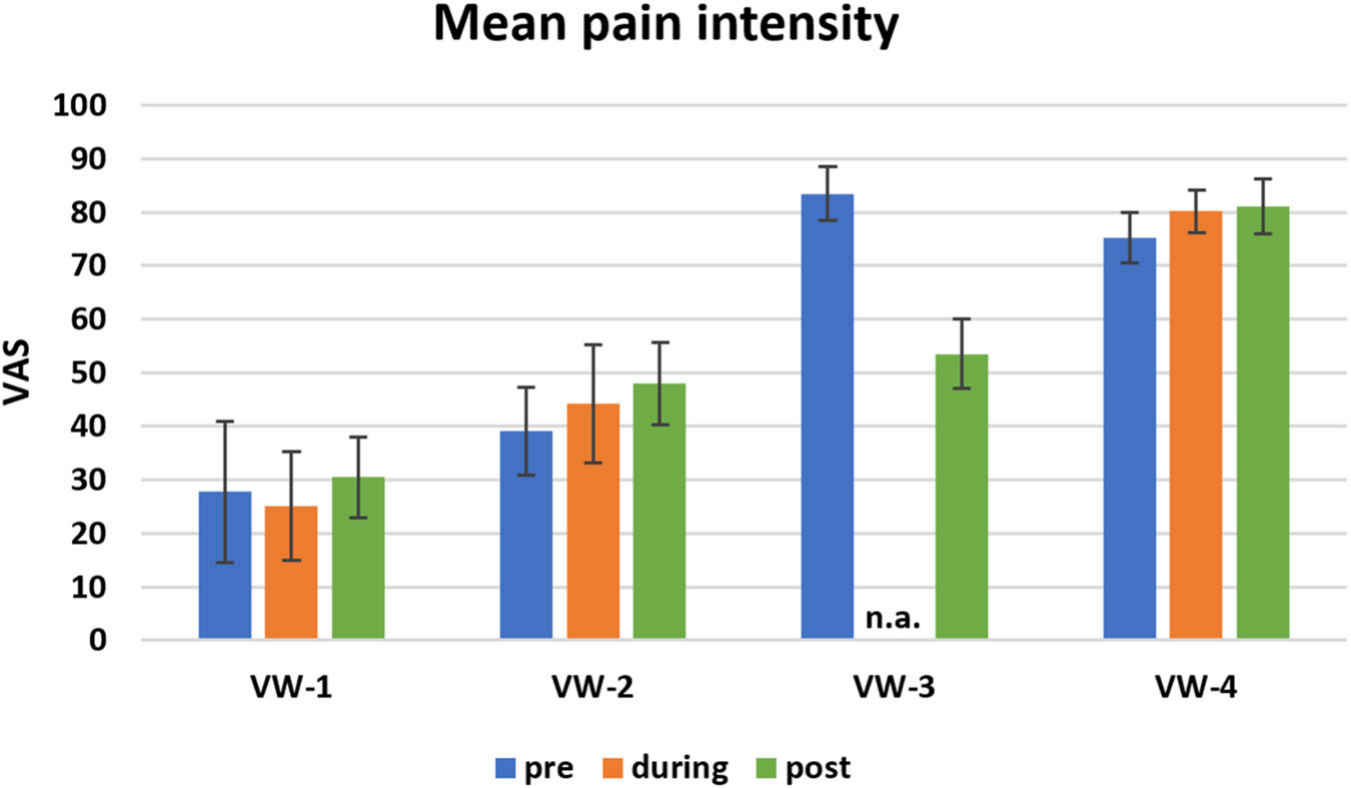
Pain intensity before, during and after virtual walking. VAS-values are shown as mean+/-SD. VAS: Visual analog scale on a 100 mm line. VW-1-4: Patient code. n.a.: not applicable due to missing values.

**Fig. 4:**
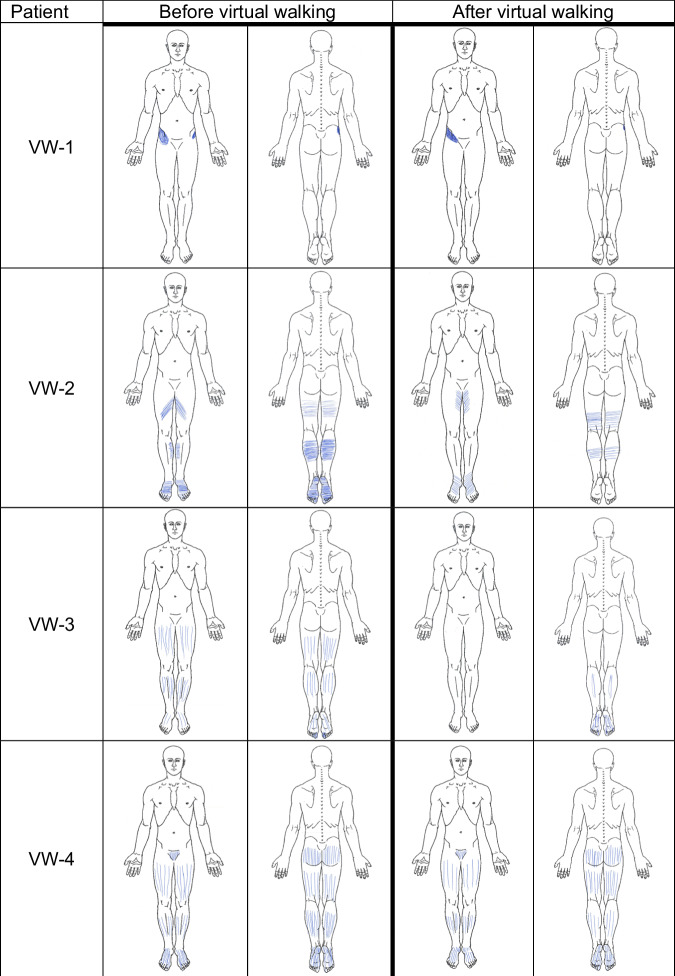
Pain drawings before and after virtual walking. Patients were asked to sketch their pain in given anatomical pictures. The time points before and after the intervention are illustrated.

Pain intensity as a secondary outcome was variable. One patient had a pain reduction by VAS 31 mm, the other three a mild clinically not relevant increase by VAS 1–7 mm (Fig. 3). One patient showed little pain improvement during VW, in one patient there were missing values during therapy. One patient reported better sleep after the therapy. Changes in pain distribution were observable in all patients. Illustrated by the pain drawings (Fig. 4). VW-1 showed only marginal to no change in pain, VW-2 showed reductions in pain in the upper limb’s ventral, the lower limb´s dorsal as well as in the foot soles. VW-3 showed the biggest reduction in pain distribution by complete cessation of pain in the leg’s ventral as well as in the foot dorsum and a big reduction in pain on upper leg's dorsal and lower legs. In VW-4 pain distribution was almost the same before and after VW therapy.

#### Pain related data and psycho-social comorbidity

MPSS showed the highest stadium of pain chronification in VW-2-4 and stadium 2 in VW-1 (questionnaires shown in Table 2).

**Table 2:** Outcomes of the self-administered questionnaires.

Assessment (range of values)	VW-1	VW-2	VW-3	VW-4
	pre | post	pre | post	pre | post	pre | post
**MPSS** stadium (1–3)	2 | n.a.	3 | n.a.	3 | n.a.	3 | n.a.
**GCPS** grade (0-4)	1 | 1	1 | 3	4 | 3	4 | 4
**PGIC** (1–7)	n.a. | 5	n.a. | 4	n.a. | 7	n.a. | 5
**DASS** (0-21)				
Anxiety	2 | 5	3 | 3	13 | 1	5 | 6
Depression	2 | 5	6 | 3	16 | 3	20 | 20
Stress	7 | 8	12 | 9	14 | 2	14 | 16
**PCS** (0-52)	16 | 11	21 | 17	29 | 8	35 | 40
**SCI-QoL-BDS** (0-10)				
Lfe	7 | 7	4 | 5	7 | 5	1 | 2
Body	6 | 7	0 | 2	7 | 6	1 | 2
Mood	8 | 8	1 | 6	6 | 6	1 | 2

*GCPS* graded chronic pain scale, *DASS* depression anxiety and stress scale, *MPSS* mainz pain staging system, *PCS* pain catastrophizing scale, *PGIC* patient global impression of change (only post); *SCI-QoL-BDS* spinal cord injury-quality of life-basic data set, *n.a.* not applicable.

VW-3 and VW-4 scored the highest grade of chronic pain severity on GCPS before therapy and VW-1 and VW-2 with lowest grade. After therapy, no changes were measured in VW1 and VW-4. VW-2 showed an increase to grade 3 and VW-3 a decrease to grade 3.

The PGIC score after treatment was 4 in VW-2, 5 in VW-1 and VW-4, and highest in VW-3 with the maximum score of 7.

DASS as a psychological outcome measure showed in the domain anxiety in VW-1 and VW-2 normal rates <6. VW-3 and VW-4 showed an increased probability of the presence of a pronounced burden of anxiety disorder before therapy. At baseline the domain of depression showed normal rates <10 in VW-1 and VW-2 and in VW-3 and VW-4 an increased probability of the presence of a pronounced burden of a depression disorder. VW-1, VW-2, and VW-4 showed no clinically relevant changes. The domain of stress showed an increased probability of the presence of a pronounced burden of a stress disorder in three patients. Only VW-1 had normal rates <10. VW-1 and VW-4 did not change after therapy whereas VW-2 changed from 12 points at baseline to 9 points after. VW-3 showed a clinical reduction from baseline in all three domains after treatment.

The PCS showed a relevant reduction of pain-related catastrophizing in three patients. However, VW-4 pointed out an increase in catastrophizing.

SCI-QoL-BDS showed no relevant changes in all patients.

## Discussion

The aim of the study was: (1) to assess the feasibility of VW in a small group of patients with SCIP in a clinical setting, (2) to investigate satisfaction with acceptance of and adherence to the therapy, and (3) to get patient’s feedback about their experiences and potential improvements of the setup.

Our VW-setting uses a green screen, a tilting wheelchair, and a natural environment on a big screen. Such a setting has not been used yet to the best of our knowledge. In some studies, a mirror was used with a video projection of walking legs [7, 15–17], in others the walking stimuli consisted of a video of an actor, in first-person view, walking along a path [18, 19] and in the last one participants viewed their virtual arms and legs in first person view in a virtual world through a head-mounted display [20].

All participants accepted the therapy well and adhered to the single sessions. There were no lasting side effects. Only one patient was more tired than usual. To date only one study [7] described side effects like distress and increased pain intensity in one patient.

In our study, there was sound satisfaction and acceptance amongst participants. Support, duration, and number of sessions were perceived well. The results indicate that the VW in our third-person perspective setting is feasible for patients with SCIP. Some of the participants were unable to fully immerse into the situation, mostly questioning the third-person perspective. In the current literature, a comparison between third-person and first-person perspective has not yet been reported. Third-person perspective has already been described [7], [15–17], while first-person perspective was also applied [18–20]. Due to different study designs and subject heterogeneity, the most helpful perspective remains an open question and depends on various additional factors.

There was a high therapy adherence in our study. Low adherence during exercise programmes in patients with SCI without pain were reported [9]. The different studies regarding VW did not discuss therapy adherence [7, 15–20].

Patient’s belief about the efficacy of treatment measured with PGIC showed a high degree also in patients who experienced a slight pain intensity increase. Similar results have been reported [20].

The patients’ feedback regarding wheelchair-related noise will be considered to improve in further studies.

In addition, pain intensity increased slightly in all, but one participant with at-level pain only. A reduction of pain distribution was shown in one participant. The participant with the best results had the longest time since SCI amongst participants and is declared as at-level pain. This finding is similar to a former study where a patient with at-level pain and the longest time since SCI also profited the most from the therapy [7, 21].

Therefore, it is actually unclear which type of SCI will most benefit from VW [21]. The mentioned patient with the best results in pain reduction showed a high decrease in all areas of the questionnaires except for the SCI-Qol-BDS which showed no change. That patient´s depression score changed significantly. A decline in depression was reported [20], however, this is the only study which examined depressive symptomatology. This study used the first-person perspective. It is known that VW in this perspective is an established tool for acute affect modulation and acute pain relief [22, 23]. Possibly different outcomes depend on the perspective. In those patients who did not respond to VW no remarkable changes in questionnaires prior and post therapy was found.

Finally, the interpretation of this current study has some limitations to consider. Due to the small sample size the findings might not be generalized for the whole SCI community. Nevertheless, there were no negative remarks or indications that the feasibility is not given.

## Conclusion

The results indicate that the therapy is feasible for patients with neuropathic pain after SCI. All participants accepted the therapy and were adherent to the single sessions. To draw conclusions about the effectiveness and therapeutic outcome on pain a further study with more patients is needed.

### Supplementary information

Supplementary Figure 1:
