# Supplementary material for: Virtual walking therapy in neuropathic spinal cord injury pain: a feasibility study
Source: Spinal Cord Ser Cases. 2024 Jul 31;10:53. doi: 10.1038/s41394-024-00667-w (PMC11289286; doi:10.1038/s41394-024-00667-w)
Supplement: Supplementary file 1 — Supplementary Figure 1: [file 41394_2024_667_MOESM1_ESM.docx]

**Supplementary figure legends:**

***Supplementary Figure 1:*** Feasibility questionnaire. English translation (not validated) of the feasibility questionnaire that was applied in German language.

**
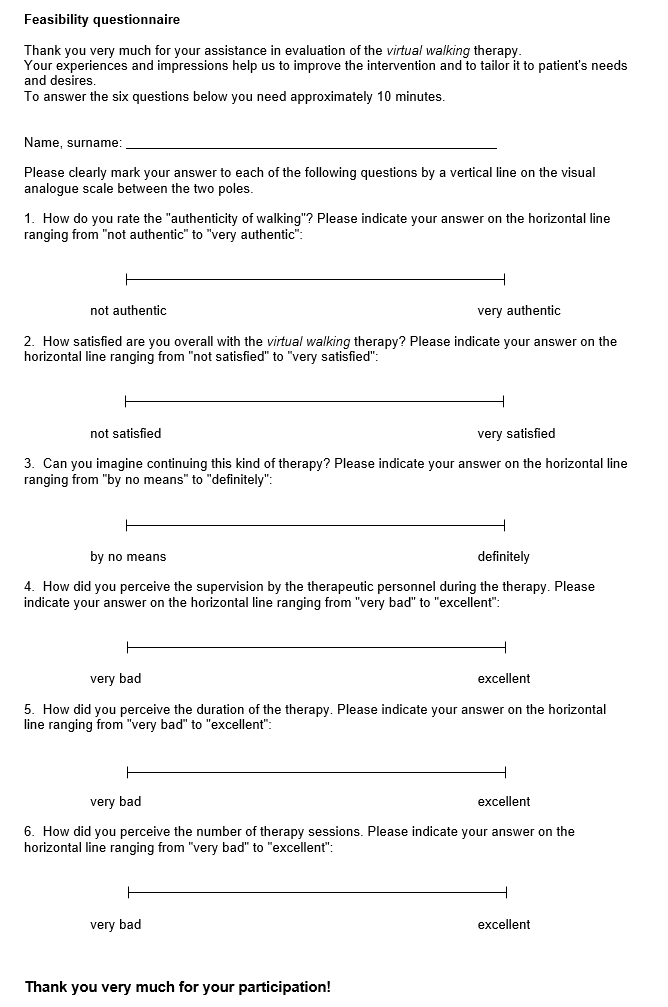
**
